# Thulium laser enucleation of the prostate plus thulium fiber laser therapy for benign prostatic hyperplasia combined with bladder stones

**DOI:** 10.20452/wiitm.2024.17897

**Published:** 2024-08-08

**Authors:** Yuanwei Li, Yongjun Yang, Junjie Chen, Zhuo Li, Guangqing Song, Jia Chen, Liu Zhe, Yili Teng, Qiang Lu

**Affiliations:** Department of Urology, Hunan Provincial People’s Hospital (First Affiliated Hospital of Hunan Normal University), Changsha, Hunan Province, China

**Keywords:** benign prostatic hyperplasia, bladder stone, thulium fiber laser, thulium laser enucleation of the prostate

## Abstract

**INTRODUCTION:**

Population aging is associated with increased incidence of benign prostatic hyperplasia (BPH), which may be complicated by bladder stones (BSs). The conditions are successfully treated with transurethral enucleation of the prostate (TUEP) plus bladder lithotomy. However, open surgery is associated with higher blood loss, larger incisions, prolonged operative time, and may impede patient recovery

**AIM:**

This work explored the feasibility, safety, and efficacy of using thulium laser enucleation of the prostate (ThuLEP) and thulium fiber laser (TFL) on a single‑energy platform to treat BPH combined with BS.

**MATERIALS AND METHODS:**

Thirty‑one patients with BPH complicated by BSs who underwent ThuLEP+TFL at our institution between October 2020 and September 2022 were included in the observation group, while 31 patients undergoing TUEP plus bladder lithotomy during the same period constituted the control group. Data collection involved assessing differences in the International Prostate Symptom Score (IPSS), hemoglobin (Hb) levels, maximum urinary flow rate (Q_max_), and quality of life (QoL) before and after surgery, along with follow‑up results.

**RESULTS:**

The patients in the observation group exhibited lower surgery duration, smaller postoperative decrease in Hb levels, shorter duration of postoperative indwelling catheterization, and proportion of American Society of Anesthesiologists (ASA) II patients, with a higher proportion of ASA I patients than the individuals in the control group (*P *<0.05). Additionally, in comparison with the control group, the patients in the observation group had lower postoperative IPSS scores and higher levels of Hb, Q_max_, and QoL score (*P *<0.05). There was no significant difference in the postoperative stone clearance rate between the 2 groups (*P *>0.05); however, the postoperative length of hospital stay was shorter in the observation group (*P *<0.05), with a higher incidence of complications in the control (*P *<0.05)

**CONCLUSIONS:**

In summary, ThuLEP+TFL surgery was safe and feasible in treating BPH+BS, relieving the symptoms, and improving QoL.

## INTRODUCTION

With accelerated population aging in China, the incidence of benign prostatic hyperplasia (BPH) among middle‑aged and elderly men is increasing annually, making it a common condition in urology outpatient clinics.^1^ Patients with BPH typically present with symptoms such as nocturia, urgency, hesitancy, and altered urine stream.^2^ These symptoms not only affect the quality of life (QoL), but may also lead to complications, such as hemorrhoids and inguinal hernias. In severe cases, they can cause bladder dysfunction, hydronephrosis, and renal failure.^3^^;^^4^Due to prostatic enlargement causing urethral obstruction and bladder dysfunction, BPH patients often develop bladder stones (BSs). Clinical statistics suggest that approximately 10% of BPH patients have concurrent BSs, presenting mainly with symptoms such as frequency, urgency, dysuria, hematuria, and interrupted urination.^5^^;^^6^ The presence of BSs in BPH patients exacerbates the risk of urinary retention and urinary tract infections, further complicating treatment. Currently, research has been conducted utilizing transurethral enucleation of the prostate (TUEP) plus bladder lithotomy for the treatment of BPH complicated by BSs, yielding favorable outcomes.^7^ However, open surgery is associated with higher blood loss, larger incisions, prolonged operative time, and may impede patient recovery.

Traditional treatment methods for BPH include medication and surgical intervention. While medication can alleviate symptoms, its efficacy is limited in patients with concurrent BSs. In terms of surgical treatment, transurethral resection of the prostate (TURP) has been historically effective in BPH management; however, it has certain limitations when addressing concurrent BSs, such as prolonged operative time, significant bleeding, and slow postoperative recovery, thereby restricting its clinical applicability. With ongoing advancements in medical technology, laser therapy has gained widespread adoption in the treatment of BPH. Since 2016, holmium laser enucleation of the prostate (HoLEP) or holmium laser enucleation of the prostate vaporization have been recommended by the European Association of Urology (EAU) guidelines as an alternative to TURP and HoLEP for transurethral prostatectomy.^8^ Since 2018, the EAU guidelines have directly recommended ThuLEP and HoLEP as first‑line treatments for large‑volume BPH.^9^ Our hospital has been performing ThuLEP since 2020 with satisfactory results. BS is a common comorbidity in BPH patients, and usually requires simultaneous surgical treatment of both conditions. Holmium: yttrium‑aluminum‑garnet (Ho:YAG) laser is considered the gold standard for ureteroscopy lithotripsy.^10^ Recently, thulium fiber laser (TFL) has been introduced as a novel technology for BS treatment, demonstrating its ability to challenge the preferred laser status of Ho:YAG for BS therapy.^11^ TFL offers higher energy density and shorter pulse width, enabling more effective stone fragmentation and tissue cutting. Compared with traditional holmium laser, TFL presents significant advantages in terms of energy transmission, surgical efficiency, and tissue preservation.

Despite numerous studies on individual applications of ThuLEP and TFL in prostate and urinary system lithotripsy, there is a scarcity of clinical research on the use of ThuLEP combined with TFL for the treatment of BPH complicated by BSs in real‑world clinical settings. Therefore, this study retrospectively analyzed the clinical efficacy and safety of ThuLEP combined with TFL for the treatment of BPH complicated by BSs at our institution from 2020 to 2022. It aimed to explore the advantages and limitations of this novel technique in clinical practice, providing scientific evidence and guidance for the treatment of similar patients in the future.

## AIM 

The aim of this study was to explore the feasibility, safety, and efficacy of ThuLEP and TFL on a single‑energy platform to treat BPH complicated by BSs.

## MATERIALS AND METHODS 

### Patients

This study was a retrospective single‑center case series. It collected data from 31 patients with BPH complicated by BSs who underwent concurrent treatment with ThuLEP+TFL at our institution from October 2020 to September 2022. This group was designated as the observation group. Simultaneously, 31 patients treated in the same period with TUEP plus bladder lithotomy were selected as the control group. The patients were evaluated by an attending physician for meeting the surgical criteria and conditions for transurethral BS fragmentation and ThuLEP. All patients signed the informed consent form, and this study obtained ethical approval from the institution. Guidelines set out in the Declaration of Helsinki were followed.

The study enrolled patients meeting the following inclusion criteria: 1) fulfilling basic requirements for surgical anesthesia assessment, with anesthesia risk rating of grade I to II (according to the American Society of Anesthesiologists [ASA] scoring system)^12^; 2) preoperative prostate‑specific antigen (PSA) determination, ultrasound, or magnetic resonance imaging, and if necessary, biopsy to rule out prostate cancer; 3) International Prostate Symptom Score (IPSS) equal to or above 12 points accompanied by QoL score of at least 4 points, or accompanied by maximum urinary flow rate (Q_max_) up to 15 ml/s, or poor response to medical treatment;^13^^;^^14^ 4) no contraindications to anesthesia or surgery; and 5) no mental illness and signing informed consent.

The exclusion criteria included: 1) uncontrolled diabetes, hypertension, cardiovascular or cerebrovascular diseases, severe liver or kidney dysfunction, or coagulation disorders; 2) uncontrolled urinary tract infection, or urethral stricture preventing completion of transurethral surgery; 3) unconfirmed prostate cancer or neurogenic bladder or neurological disorders affecting urinary function; and 4) a history of lower abdominal surgery.

### Surgical methods

The patients in the control group underwent TUEP plus bladder lithotomy. The surgery was performed under combined spinal‑epidural anesthesia with continuous monitoring of vital signs. During BS extraction, a resectoscope was inserted through the urethra to visualize the BSs and assess the extent of prostatic enlargement. Upon confirming the inability to retrieve the stones transurethrally, TUEP was conducted using continuous irrigation and flushing. Following completion of the  resection, a triple‑lumen urinary catheter was left in place, and the bladder was continuously irrigated with 0.9% saline. During the procedure, a midline abdominal incision was made approximately 12 cm above the symphysis pubis, with a length of 34 cm. The incision involved the skin, subcutaneous tissue, anterior sheath of the rectus abdominis muscle, and separation of the rectus abdominis muscle. The peritoneum was then reflected upwards, exposing the bladder. The anterior bladder wall was incised, and stones were retrieved under direct visual control. After confirming no residual blood clots or significant bleeding in the bladder, the bladder was sutured. Intermittent plasma drainage was performed anteriorly to the bladder without the need for cystostomy. The incision was closed layer by layer, and a triple‑lumen urinary catheter was left in place postoperatively, with continuous bladder irrigation.

The patients in the observation group were treated with ThuLEP+TFL. The patients were placed in the lithotomy position and administered general or epidural anesthesia. A fiber‑optic holmium laser (RevoLix, Shanghai, China) was used with cutting or vaporization power settings of 60–120 W and hemostasis power settings of 20–40 W. The fragmentation mode was set according to the hardness of the stone, with energy ranging from 0.1 to 2 J and power from 30 to 60 W. A Fr26 laser resection sheath with a continuous flushing system and a reusable fiber with a diameter of 550 µm were used for stone fragmentation and tissue vaporization. Physiological saline was employed for continuous irrigation during the procedure, with a flow rate of 50–100 ml/min through the resection sheath. The surgery was performed by experienced surgeons. Stone fragmentation first involved a powdering strategy, and the stone powder was continuously flushed out through the lens body. When the remaining stone volume was smaller than the sheath, it was washed out through the sheath. After the fragmentation, the urethral mucosa was cut open in an omega shape in front of the verumontanum, and the surgical capsule plane of the prostate was found using the “push and pull” technique with the resection sheath. The entire prostate adenoma was then completely peeled off along the surgical capsule plane under direct visualization of the laser resection sheath. The holmium laser was utilized to sever the connection between the prostate adenoma and the urethral mucosa at the bladder neck and apex of the prostate, to vaporize the prostate nodules on the surgical capsule plane, and to fully stop bleeding at the bleeding points on the surgical capsule plane and the bladder neck. The excised adenoma was pushed into the bladder, and the external sphincter and bladder neck were completely preserved. Finally, a tissue pulverizer was utilized to crush the glandular tissue, which was then removed from the body, and the tissue sample was saved for pathological examination. A Fr20 3‑chamber balloon catheter was left in place.

### Evaluation indicators

Before surgery, we collected data on all patients, including their age, comorbidities, number of stones, serum PSA level, prostate volume, stone burden (calculated based on the widest diameter of a single stone or as a sum of the widest diameters of all stones), average stone density (determined by computed tomography [CT] scan), preoperative blood routine (hemoglobin [Hb] and white blood cell count), pre‑operative IPSS, QoL score, and Q_max_.

The perioperative indicators included operation time, duration of lithotripsy, duration of bladder irrigation, Hb decrease, postoperative duration of catheterization, and ASA classification.

The therapeutic efficacy was assessed by evaluating the difference in IPSS score, Hb level, Q_max_, and QoL before and after the surgery.

The postoperative follow‑up indicators included length of hospital stay, daily phone conversation or internet follow‑up within 1 week after discharge, instructing the patient to undergo regular check‑ups, and evaluating the stone clearance, bleeding, infection, urinary retention, perforation, postoperative urethral stricture, and other complications based on CT scans.

### Statistical analysis

The data were analyzed using SPSS package version 23.0 (IBM, Armonk, New York, United States). Normally distributed quantitative data were presented as mean (SD), while non‑normally distributed data were expressed as percentage. The Wilcoxon rank‑sum test was utilized to compare functional data before and after surgery, with a P value below 0.05 indicating statistical significance.

## RESULTS

### General patient data

The general clinical characteristics showed in Table 1 indicated no significant differences in the general clinical indicators between the 2 groups before the surgery.

### Comparison of the perioperative conditions

The study collected data on surgical duration, stone fragmentation/ extraction time, postoperative decrease in Hb levels, bladder irrigation time, duration of postoperative urinary catheterization, and ASA score grading Table 2. As compared with the control group, the patients in the observation group exhibited shorter surgery duration, smaller postoperative decrease in Hb levels, shorter duration of postoperative urinary catheterization, and lower proportion of ASA II grade, with a higher proportion of ASA I grade (*P *<0.05). However, there were no significant differences in stone fragmentation/ extraction time or bladder irrigation time between the 2 groups.

### Surgical treatment efficacy

**Figure 1:**
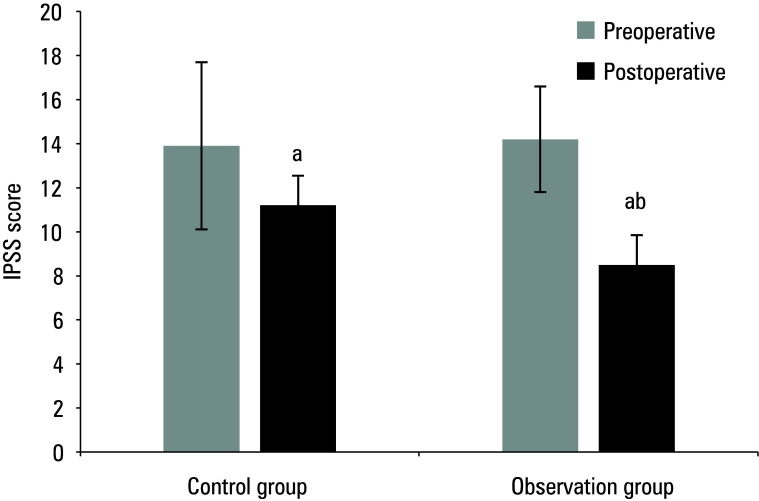
Mean International Prostate Symptom Score (IPSS) before and after surgery

**Figure 2:**
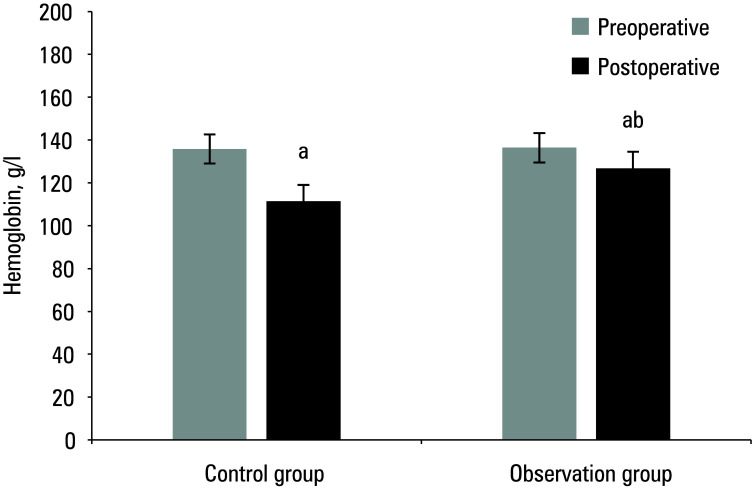
Mean hemoglobin levels before and after surgery

**Figure 3:**
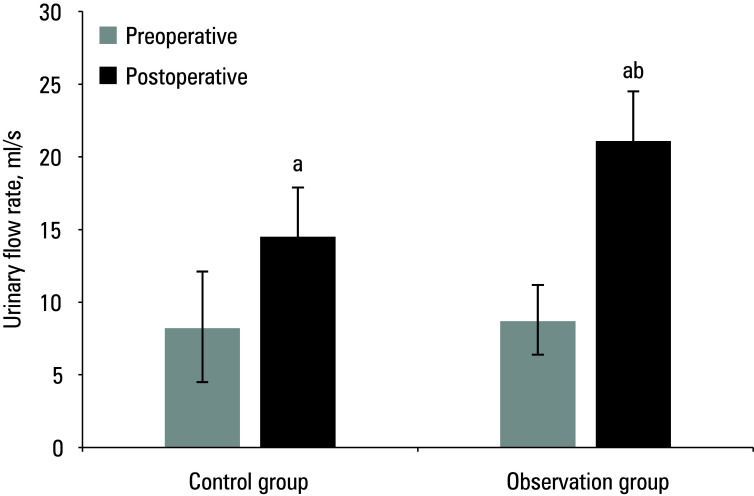
Mean maximum urinary flow rate before and after surgery

**Figure 4:**
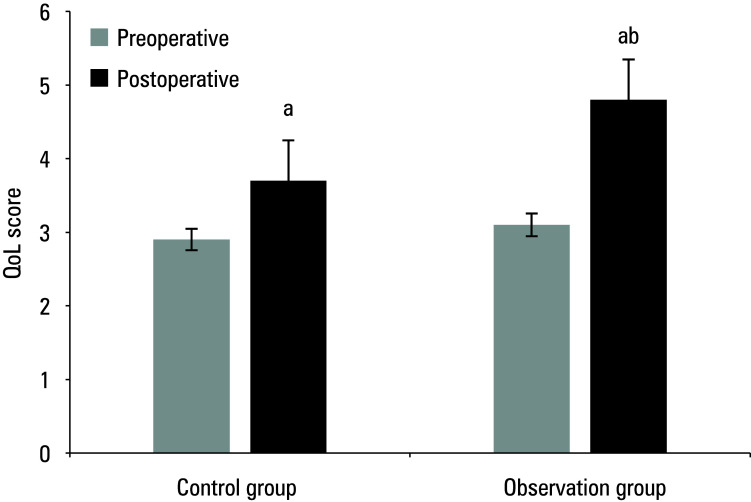
Mean quality of life (QoL) score before and after surgery a Significant difference as compared with preoperative values, P <0.05 b Significant difference as compared with the control group, P <0.05

The study compared the pre and post‑treatment IPSS, Hb levels, Q_max_, and QoL in the 2 groups of patients. Preoperative results are presented in Table 1**.** Mean (SD) postoperative IPSS score, Hb level, Q_max_, and QoL score for the control group were 11.2 (3.2) points, 111.4 (10.8) g/dl, 14.6 (9.3) ml/s, and 3.7 (0.4) points, respectively, while for the observation group, they were 8.5 (2.4) points, 126.4 (15.9) g/dl, 21.2 (13.7) ml/s, and 4.8 (0.4) points, respectively. As compared with the preoperative values, both groups showed a postoperative decrease in IPSS score and Hb levels, and an increase in Q_max_ and QoL score (*P* <0.05). The patients in the observation group exhibited lower postoperative IPSS scores and higher Hb levels, Qmax, and QoL scores (*P *<0.05) Figure 1^;^Figure 2^;^Figure 3^;^Figure 4.

**Table 1:** Preoperative general characteristics of the patients

Variable	Observation group (n = 31)	Control group (n = 31)	*P *value
Age, y	67.9 (7.8)	65.1 (6.2)	0.24
Comorbidities, n (%)			
Hypertension	13 (41.9)	12 (38.71)	0.16
Diabetes	5 (16.1)	6 (19.35)	
Coronary artery disease	4 (12.9)	5 (16.13)	
Myocardial infarction	2 (6.5)	3 (9.68)	
Cerebral infarction	2 (6.5)	4 (12.9)	
Urinary tract infection	3 (9.7)	2 (6.45)	
Other	12 (38.7)	10 (32.26)	
Number of stones, n (%)			
1	11 (35.5)	13 (41.94)	0.1
2	6 (19.4)	5 (16.13)	
≥3	14 (45.2)	13 (41.94)	
Serum PSA, ng/ml	5.5 (5.2)	5.3 (5)	0.09
Prostate volume, ml	64.1 (43.4)	62.8 (36.2)	0.11
Stone burden, mm	21.9 (11.6)	22.6 (9.2)	0.19
Mean stone density, HU	759.2 (300)	737.9 (273.2)	0.1
Preoperative Hb, g/l	136.4 (15.9)	135.8 (13.8)	0.11
Preoperative white blood cell count, 10^9^ /l	7.6 (3.3)	7.2 (3.4)	0.07
Preoperative IPSS score, points	14.2 (3.8)	13.9 (3.2)	0.07
QoL score, points	3.1 (0.9)	2.9 (0.8)	0.08
Maximum flow rate, ml/s	8.8 (6.2)	8.3 (5.3)	0.11

Data are presented as means (SD). SI conversion factors: to convert hemoglobin to g/l, multiply by 10. Abbreviations: IPSS, International Prostate Symptom Score; PSA, prostate‑specific antigen; QoL, quality of life

**Table 2:** Perioperative characteristics of the patients

Variable	Observation group (n = 31)	Control group (n = 31)	*P *value
Surgery duration, min	109 (29.1)	130.4 (20.3)	0.02
Stone fragmentation / extraction time, min	34.7 (27.6)	30.8 (22.3)	0.052
Postoperative decrease in Hb levels, g/dl	0.1 (0.2)	0.3 (0.1)	0.001
Bladder irrigation time, d	2.1 (0.9)	1.9 (0.6)	0.06
Duration of postoperative urinary catheterization, d	5.5 (1.3)	7.3 (1)	0.02
ASA score grading, n (%)			
Grade I	19 (32.3)	13 (41.95)	0.002
Grade II	12 (29)	18 (58.06)	

Data are presented as means (SD). Abbreviations: ASA, American Society of Anesthesiologists

### Postoperative follow‑up

**Figure 5:**
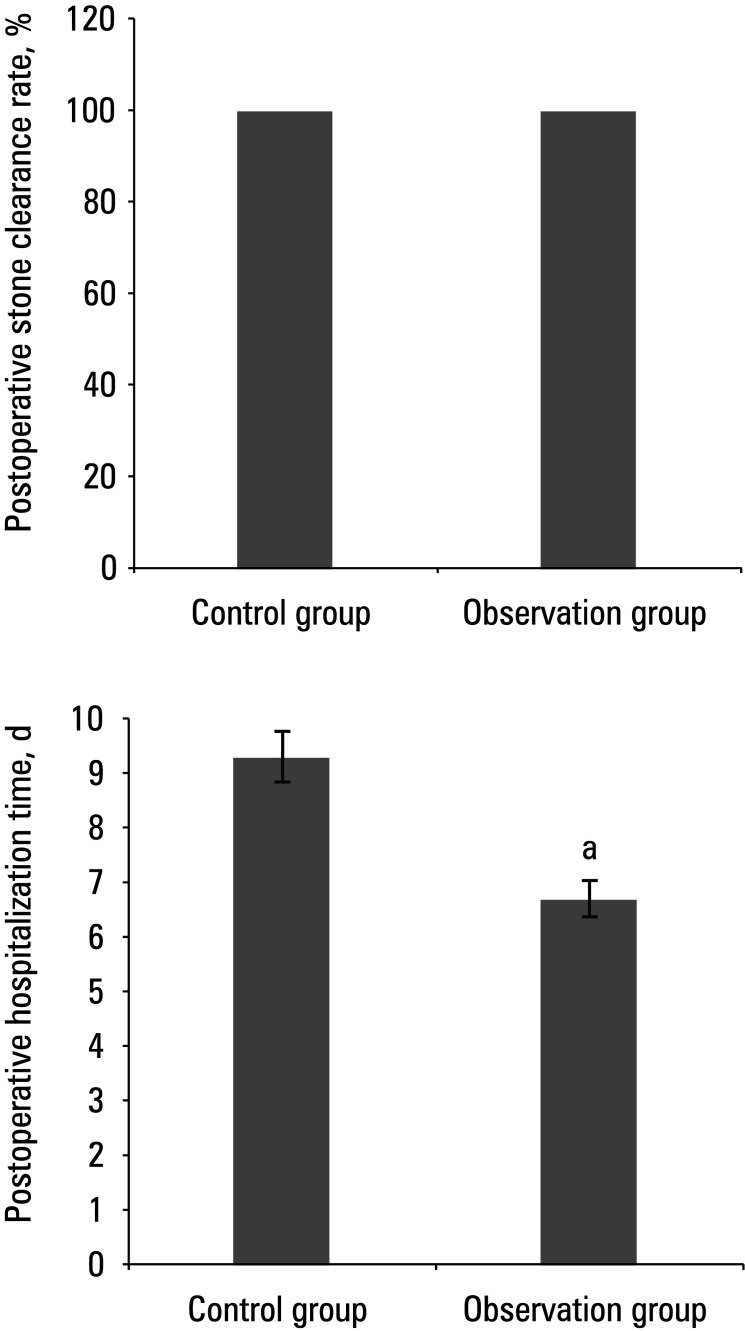
Comparison of postoperative stone clearance rate (A) and mean hospital stay (B)

All patients underwent postoperative abdominal radiography, with a 100% stone clearance rate reached in both groups. The mean (SD) postoperative hospital stay was 9.3 (3.25) days for the control group and 6.7 (2.5) days for the observation group (*P *<0.05). There was no significant difference in the postoperative stone clearance rate between the 2 groups Figure 5 During follow‑up, the patients in the observation group did not report any significant postoperative complications, whereas in the control group, there were 4 (12.9%) infections and 5 (16.13%) cases of urethral strictures and other complications (P <0.05).

## DISCUSSION

The study demonstrated superior surgical outcomes of ThuLEP combined with TFL over TUEP combined with bladder lithotomy in the treatment of BPH complicated by BSs. Additionally, the patients in the observation group had a shorter postoperative hospital stay and no severe complications. Therefore, ThuLEP combined with TFL can be considered an effective and safe surgical approach for the treatment of BPH complicated by BSs.

Firstly, the study found that the postoperative stone clearance rate was 100% in both the control and observation groups, indicating that TUEP combined with bladder lithotomy and ThuLEP combined with TFL are highly effective in clearing BSs. This may be attributed to the high‑energy and precise cutting and fragmentation capabilities of the thulium laser, which effectively clears stones from the bladder. Traditional surgical methods involve direct incision of the bladder wall, allowing for the removal of all stones under direct visual control. These findings are consistent with previous research.^15^ Our study also found no significant difference in stone fragmentation/ extraction time between the 2 groups, suggesting that TFL achieves similar efficiency in stone fragmentation as bladder wall incision for stone extraction. This is because TFL fragmentation not only induces a photothermal effect but also triggers a water explosion effect around the stones. Moreover, TFL pulse waves are square waves, allowing for more uniform energy transfer to the stones, leading to efficient stone fragmentation. Research showed that TFL‑induced stone displacement is minimal, reducing the probability of interrupted stone fragmentation, thus shortening the procedure time while achieving precise stone fragmentation.^16^^;^^17^ Therefore, the patients in the observation group did not experience significant complications that would affect subsequent ThuLEP surgery.

**a** Significant difference as compared with preoperative values, *P* <⁠0.05

**b** Significant difference as compared with the control group, *P* <⁠0.05

**a** Significant difference as compared with preoperative values, *P* <⁠0.05

**b** Significant difference as compared with the control group, *P* <⁠0.05

**a** Significant difference as compared with preoperative values, *P* <⁠0.05

**b** Significant difference as compared with the control group, *P* <⁠0.05

**a** Significant difference as compared with preoperative values, *P* <⁠0.05

**b** Significant difference as compared with the control group, *P* <⁠0.05

**a** Significant difference as compared with the control group, *P* <⁠0.05

ThuLEP utilizes the blunted dissection effect of the laser sheath to mimic the surgical plane of anatomical dissection performed with fingers during open prostatectomy, aiming to completely detach the hypertrophic adenoma from the surgical capsule, thus maximizing the relief of bladder outlet obstruction, alleviating lower urinary tract symptoms, and effectively preventing long‑term recurrence of BPH.^18^ The TFL used in this study, unlike conventional solid‑state holmium lasers, employs a fusion wave technology that combines continuous and pulse waves, ensuring that tissue temperature remains below 100 °C throughout the laser application, thereby further minimizing thermal stimulation to the bladder mucosa and the prostate bed.^19^ Additionally, it can generate a larger area of umbrella‑like hemostasis, leading to faster hemostasis and contributing to improved surgical efficiency of the ThuLEP procedure. This study also found that patients in the observation group had shorter surgical duration and smaller postoperative decrease in Hb levels. TUEP involves insertion of an electric resection loop through the urethra to excise hypertrophic prostatic tissue, thereby alleviating urinary obstruction symptoms, and is currently a mature technology.^20^ However, TUEP is associated with relatively higher levels of bleeding, often necessitating stricter intraoperative and postoperative bleeding control measures. Moreover, due to the larger trauma incurred, longer postoperative recovery times and comparatively prolonged indwelling catheter durations are often required.^21^ Additionally, studies noted that TUEP may pose a higher risk of postoperative complications, such as urethral stricture and infection.^22^ Therefore, patients in the control group were more likely to experience postoperative infections and urethral strictures, indirectly leading to lower Q_max _than in the observation group. Given that the holmium laser technology entails less tissue trauma than traditional incisional surgery, faster tissue healing, reduced postoperative bleeding, and decreased bladder irritation, postoperative recovery is quickened and catheter can be removed earlier. Consequently, the patients in the observation group had shorter postoperative hospital stay and indwelling catheter duration. The study also found that the patients in the observation group had lower postoperative IPSS and higher QoL scores. IPSS is widely used to assess the severity of symptoms in patients with BPH, and its decrease indicates effectiveness of the surgery in alleviating symptoms. This suggests that the ThuLEP combined with TFL surgical approach is more effective in improving patient symptoms. Specifically, it limits poor sleep, anxiety, embarrassment, and discomfort caused by BPH symptoms, thereby enhancing the QoL.

In summary, ThuLEP+TFL demonstrated advantages in treating BPH with concurrent BSs by shortening the procedure duration and postoperative recovery, reducing bleeding and complication rate, and improving treatment efficacy and QoL.

## CONCLUSIONS

This work reported, for the first time, the real‑world clinical application data of simultaneous ThuLEP and TFL surgery for treating BPH with concurrent BS. The preliminary results indicated that simultaneous ThuLEP and TFL surgery was a safe and feasible treatment for BPH+BS, which can effectively alleviate the symptoms of patients and improve their QoL. However, the sample size enrolled was relatively small, the follow‑up time was short, and it was a single‑center study, which may have biased the results. Therefore, further studies with increased sample size are needed to confirm these findings. In summary, the treatment approach encompassing ThuLEP and TFL is worth promoting in hospitals with appropriate conditions.
